# A successful endovascular aortic repair of aortoesophageal fistula following esophagectomy: a case report and literature review

**DOI:** 10.1186/s13019-024-02540-1

**Published:** 2024-02-07

**Authors:** Jina Li, Yan Hu, Wenliang Liu, Jingqun Tang, Song Zhu, Chao Zeng

**Affiliations:** 1 Department of Thoracic Surgery, The Second Xiangya Hospital, Central South University, Changsha, Hunan People’s Republic of China; 2 Clinical Nursing Teaching and Research Section, The Second Xiangya Hospital, Central South University, Changsha, Hunan People’s Republic of China

**Keywords:** Aortoesophageal fistula, Esophagectomy, Esophageal carcinoma, Thoracic endovascular aortic repair

## Abstract

**Background:**

Aortoesophageal fistula (AEF) is an extremely rare and highly fatal complication leading to a high risk of morbidity and mortality. Successful management of AEF after esophagectomy for esophageal carcinoma has rarely been reported in the literature.

**Case presentation:**

Here we present a rare case of a 44-year-old female with complications of AEF after esophagectomy for esophageal carcinoma, mainly presented as vomiting of blood. Both computed tomographic and computed tomography angiography of the chest showed bilateral pleural effusion and atelectasis, while gastroscopy showed large gastrointestinal bleeding. Emergency surgery was performed that included the removal of the mediastinal abscess, left lower pulmonary wedge resection, and thoracic endovascular aortic repair (TEVAR), followed by supportive treatment. The surgery went successful, and the patient was followed up for 1 year after discharge and showed good recovery. We also reviewed previous literature on the history, causes, pathophysiology, clinical presentation, diagnosis, and treatment of AEF after esophagectomy for esophageal adenocarcinoma.

**Conclusions:**

In our case, thoracotomy combined with TEVAR was effective in treating AEF after esophagectomy for esophageal adenocarcinoma. This case provides successful experiences for clinical diagnosis and treatment of AEF after esophagectomy for esophageal carcinoma.

## Background

Aortoesophageal fistula (AEF), defined as a fistula between the thoracic aorta and the esophagus, is an extremely rare and highly fatal complication that usually occurs in aortic or esophageal diseases, such as thoracic aortic aneurysm and esophageal malignant tumor, or foreign body ingestion, or after previous thoracic aortic surgery [1, 2]. AEF remains as a life-threatening emergent condition with a poor prognosis and is associated with a high risk of morbidity and mortality. AEF after esophagectomy is a rare condition that progresses very fast and often causes death within a short period of time if not treated promptly and effectively. Successful management of AEF after esophagectomy has rarely been reported in the literature. This paper herein presents the clinical presentation and successful treatment of AEF in a patient who has undergone esophagectomy for esophageal carcinoma as well as a literature review of previously reported cases of AEF.

## Case presentation

**Fig. 1:**
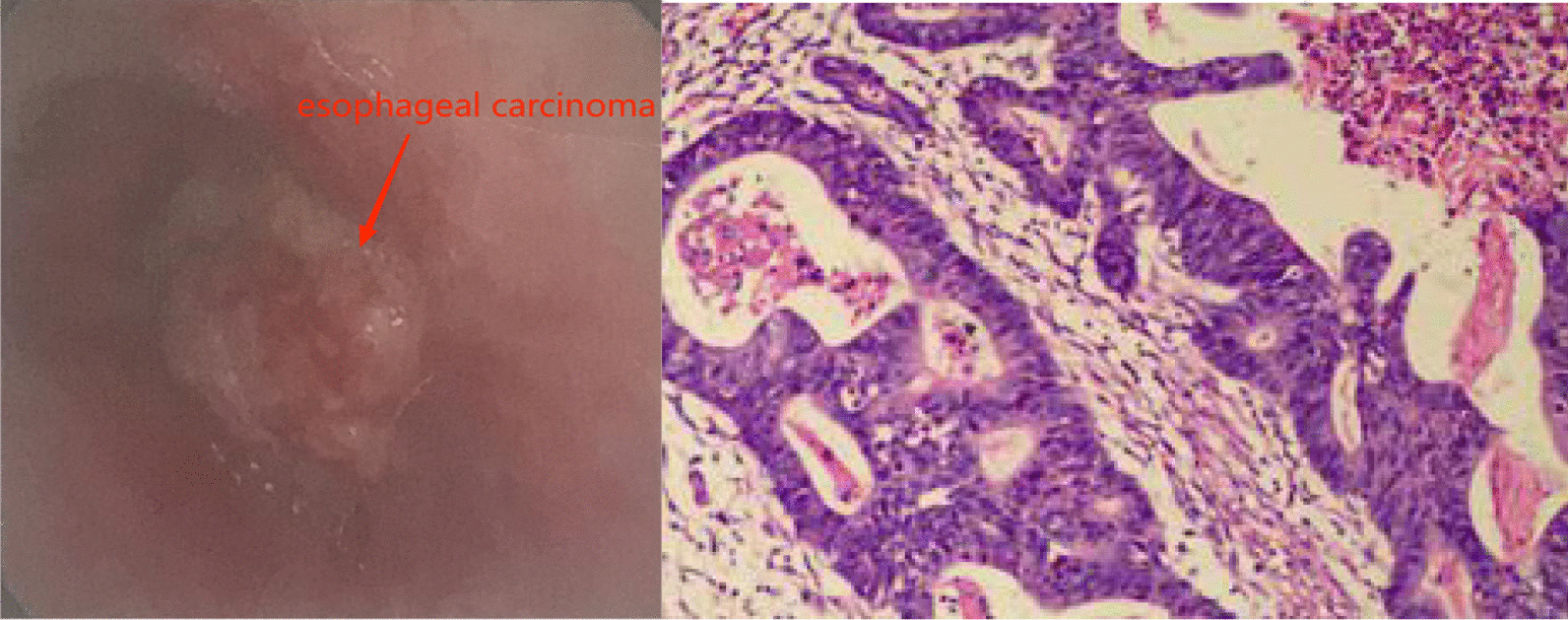
Results of preoperative gastroscope and biopsy

**Fig. 2:**
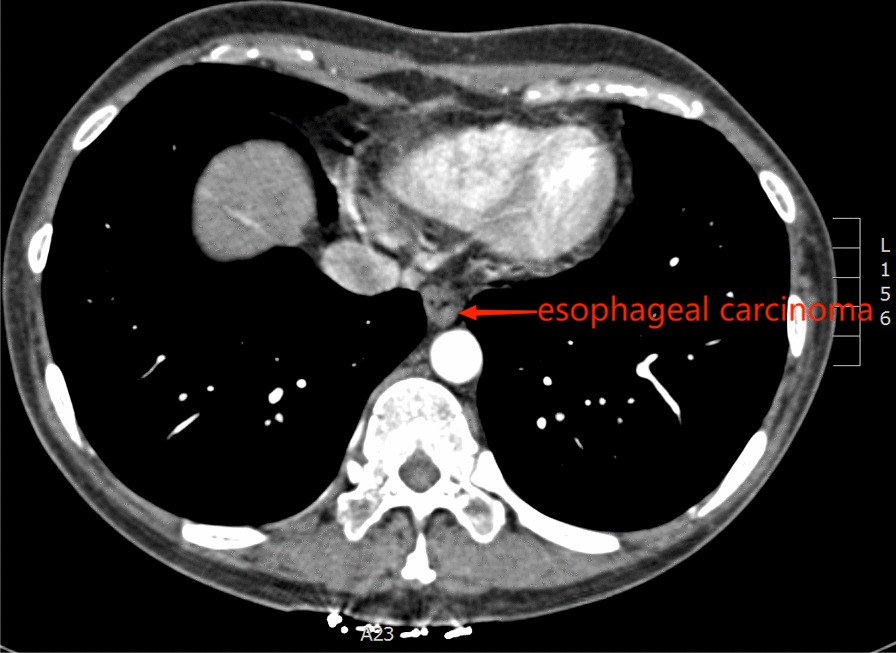
Chest CT before the operation of esophageal carcinoma

**Fig. 3:**
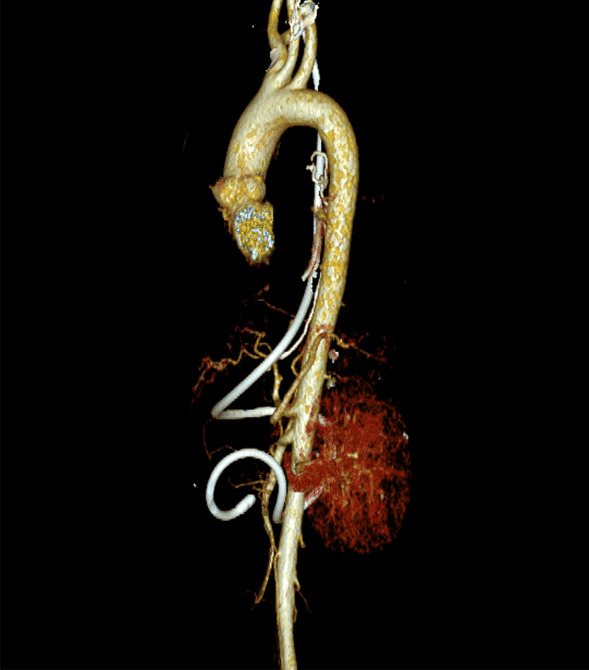
CTA of the whole aorta on the 19th day

A 44-year-old female with a main complaint of “progressive dysphagia” for over 1 month was admitted to our hospital and diagnosed with esophageal adenocarcinoma by gastroscopic biopsy (Fig. 1).Chest CT indicates that the tumor is located in the lower segment of the esophagus, 34 cm from the incisor teeth (Fig. 2).A combined radical esophagectomy and stapled esophagogastric anastomosis were performed through a left thoracotomy approach on August 9, 2019. No obvious anastomotic leakage was found in the upper gastrointestinal angiography 1-week after exploratory thoracotomy. Dynamic blood test indicates that WBC is slightly higher than normal, with a maximum of approximately 11.37 × 10^9^/L, Procalcitonin and CRP are within the normal range. However, a small amount of turbid fluid was continuously drained from the mediastinal drainage tube. Computed tomographic (CT) scans of the chest showed bilateral pleural effusion and atelectasis. Esophageal content of pink-tinged fluids was obtained through the nasal tube. We suspected an AEF developed due to the esophagectomy and performed pleural puncture and closed thoracic drainage with 400 ml of fluids drained on the 8th postoperative day. And on the 19th postoperative day, the patient suddenly vomited about 600 ml of blood and blood clot and was transferred to thoracic surgery intensive care units (ICU). The complete computed tomography angiography (CTA) of the whole aorta in the emergency department showed that the pleural effusion on both sides increased than before, with no obvious abnormalities in the total trunk of the head arm trunk and the left neck (Fig. 3). Gastroscopy showed a large amount of blood and blood clots in the feeding tube, and the mediastinal drainage tube protruded into the lumen. After a series of conservative treatments including blood transfusion, hemostasis, anti-infection, stomach protection, fasting, and rehydration, the patient showed no significant improvement, with continuously declining hemoglobin and hematocrit.

**Fig. 4:**
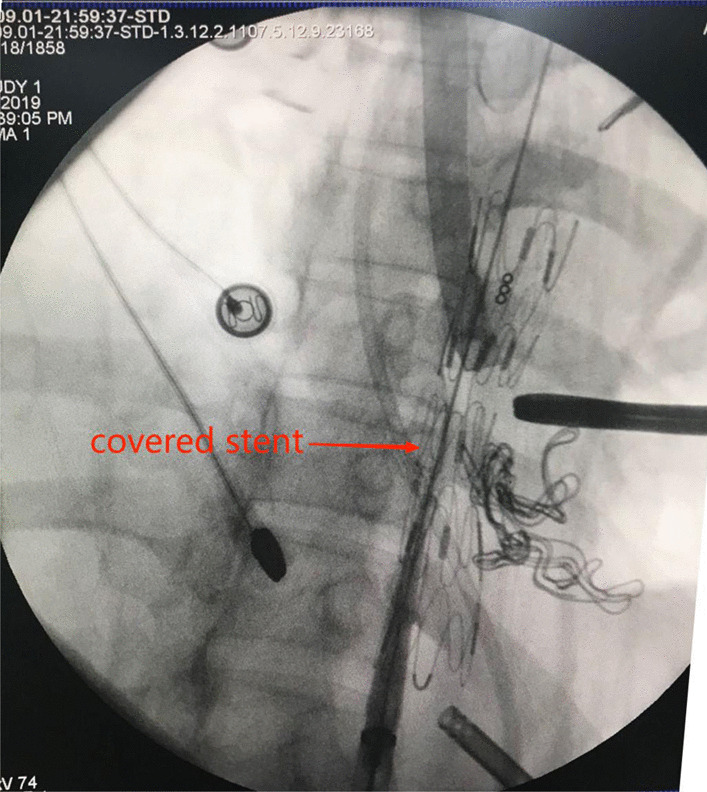
After exploratory thoracotomy and TEVAR (X-ray)

**Fig. 5:**
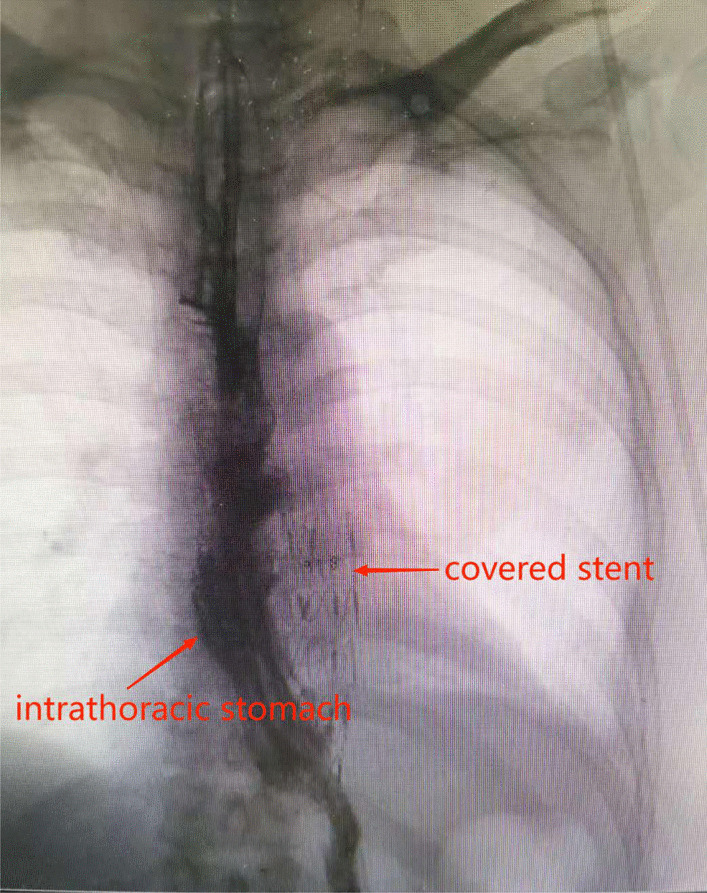
One-week after exploratory thoracotomy and TEVAR (Upper gastrointestinal radiography)

**Fig. 6:**
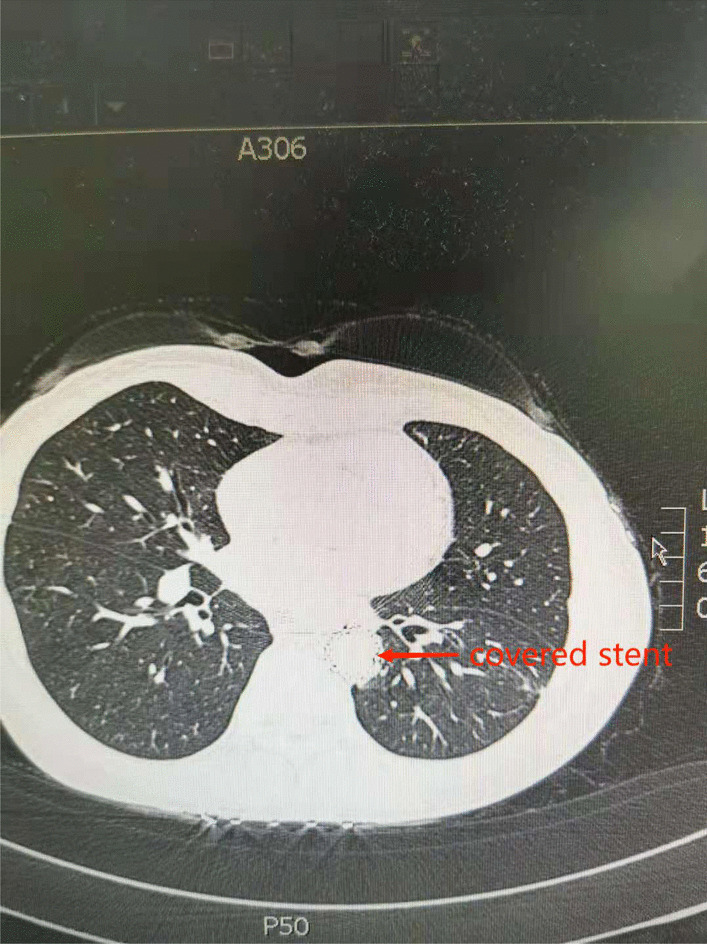
One-year after exploratory thoracotomy and TEVAR (Chest CT scan)

On the 22nd postoperative day, the patient vomited 600 ml of blood, together with the stomach tube and duodenal nutrition tube. We decided to perform emergency surgery and a laparotomy under general anesthesia was initiated on the 23rd day. The surgery included the removal of the mediastinal abscess, left lower pulmonary wedge resection, and TEVAR. During the surgery, an encapsulated purulent cavity was observed that was formed by the close adhesion of the left lower lung, pericardium, diaphragmatic muscle, and aorta. A 4 * 4 * 3 cm pus cavity was also observed in the basal segment of the left lower lung. After removal of mediastinal pus and old blood clots, a large amount of bright red blood spurted out from the level of the thoracic aortic septum, confirming our previous suspected diagnosis of AEF. Due to the heavy adhesion among the aorta, the tubular stomach, and the mediastinum, we cannot continue the repair of the aortic fistula but performed TEVAR. However, after consultation with the vascular surgical group, a femoral artery puncture was performed and a covered stent was used to successfully repair the aortic fistula. The surgery went successful: the bleeding stopped, the left lower lung abscess was removed, and the thoracic cavity was washed repeatedly with iodine and normal saline (Fig. 4). After the surgery, the patient was given supportive treatment including anti-infection, fasting, enteral nutrition, and intravenous fluid infusion, and recovered smoothly (Fig. 5). The patient was followed up for 1 year after discharge and showed good recovery with unobstructed semi-fluid intake and good stent position and function. No mediastinal infection, pleural effusion, anastomotic stoma, thoracic gastric fistula, or aortic fistula was reported since then (Fig. 6).

## Discussion

Aortoesophageal fistula (AEF) is a relatively rare but highly fatal condition that may lead to a series of life-threatening complications [3, 4]. The first identified case of AEF was reported by Dubrueil in 1818 which was caused by ingestion of a foreign body [5]. It was not until 1969, over one century after the first identified case of AEF, that the first successful treatment by surgical correction was reported [6]. Subsequently, increasing successful treatment cases have been reported in the literature [7–11].

The underlying causes of AEF are multiple, and the primary causes of AEF mainly include ruptured aortic aneurysm, foreign body ingestion, endograft stenting of the thoracic aorta, advanced esophageal cancer, or surgical procedures involving the esophagus [1]. Thoracic aortic surgery has been well recognized as a major cause of secondary AEF. In a recent review conducted by Takeno et al. [2] on 150 patients of AEF, postoperative status for aortic disease constituted the most common cause of AEF with 61 identified cases, followed by a primary aortic aneurysm (n = 45), foreign body ingestion (n = 25), and thoracic cancer (n = 23).It remains unknown the exact mechanism of AEF, though many pathophysiological mechanisms have been speculated. Some proposed hypotheses of the pathogenesis include esophageal ischemia caused by esophageal artery blockage, inflammation of the resorption hematoma, inflammation of the aneurysmal wall, increased pressure in the posterior mediastinum, mechanical compression by a large aneurysm and secondary erosion, radial force of the graft against the native aortic wall, etc. [12].

The classical clinical symptoms of AEF were first defined by Chiari in 1914 that included middle chest pain, dysphagia and sentinel hemorrhage followed by exsanguinating hematemesis, also known as Chiari triad [13]. The most common symptom of AEF after esophagectomy for esophageal carcinoma is gastrointestinal bleeding, also known as signal hematemesis. The incidence of critical gastrointestinal bleeding due to AEF was reported to be 5–23% by a meta-analysis [14]. The amount of bleeding varies from hundreds of milliliters to thousands of milliliters and lasts from several hours to several days. Bleeding can be sustained, fluctuating, or sudden fatal bleeding. Apart from typical clinical symptoms, the diagnosis of AEF can be supported by a chest radiograph showing mediastinum enlargement, and an endoscopic examination revealing esophageal wall mass covered by adherent blood clots or with active hemorrhaging that confirms the source of bleeding. A further CT or digital subtraction angiography (DSA) can be used to confirm the diagnosis when there is active bleeding of ≥ 0.5 ml/min [8, 15].

Surgical therapy was primarily used as a major treatment of AEF, with several alternative strategies being reported in the literature that included extra-anatomic bypass and in situ repair with cryopreserved homograft [16]. Due to the rare condition of AEF and lack of large sample data, the prognosis of surgical management of AEF remains largely unknown and no consensus has been reached concerning the optimal treatment for AEF. However, the clinical outcomes have significantly improved due to the popularization of endoluminal aortic stent therapy, especially thoracic endovascular aortic repair (TEVAR). TEVAR was developed recently as a minimally invasive therapy for AEF and has shown success in treating thoracic aortic aneurysms and other thoracic aortic pathologies [17–20]. Although less invasive, TEVAR also has its own limitations in treating AEF, which are mainly caused by the risk of graft contamination [16]. As a result, TEVAR has been proposed to be bridge therapy for hemostasis as an alternative damage-control surgery to surgical management [21–24].

Several combinations have been proposed that involved TEVAR with surgical aortic replacement, esophageal reconstruction after esophagectomy, or mediastinal drainage [25–27]. A review conducted by Akashi et al. [16] showed that the midterm survival of AEF was significantly improved by esophagectomy, open surgery with aortic replacement using prostheses and homografts, and greater omentum wrapping. Another recent review by Takeno et al. [2] also showed that a combination of surgery for the aorta (TEVAR, graft replacement or repair) and esophagus (esophagectomy, esophageal stent or repair) was associated with a favorable prognosis, with the combination of graft replacement and esophagectomy resulting in the most favorable prognosis of all therapies. Apart from surgical treatment, antibiotic treatment is also widely proposed as a supportive treatment for AEF [2]. AEF is actually an infection of the bacteria-free aorta caused by contamination from the gastrointestinal tract material. As a result, the use of strong, broad-spectrum antibiotics is effective in the prevention of post-surgery sepsis and should be considered in the treatment of AEF.

## Conclusions

This paper reports the clinical presentation and successful management of a rare case of AEF after esophagectomy for esophageal carcinoma. We also review previous literature on the history, causes, pathophysiology, clinical presentation, diagnosis, and treatment of AEF. AEF is an extremely rare and highly lethal condition associated with a series of life-threatening complications that may lead to a high risk of morbidity and mortality. Successful management relies on timely diagnosis, early intervention, and a combination of surgery and supportive treatment.

## Abbreviations

AEF: Aortoesophageal fistula
TEVAR: Thoracic endovascular aortic repair
CT: Computed tomographic
ICU: Intensive care unit
CTA: Computed tomography angiography
DSA: Digital subtraction angiography
